# A Biopredictive ***In Vitro*** Approach for Assessing Compatibility of a Novel Pediatric Hydrocortisone Drug Product within Common Pediatric Dosing Vehicles

**DOI:** 10.1007/s11095-020-02912-x

**Published:** 2020-09-24

**Authors:** Erik Wollmer, Frank Karkossa, Lisa Freerks, Anna-Elena Hetberg, Greg Neal, John Porter, Martin J. Whitaker, Daniel Margetson, Sandra Klein

**Affiliations:** 1 Department of Pharmacy, Institute of Biopharmaceutics and Pharmaceutical Technology, Center of Drug Absorption and Transport (C_DAT), University of Greifswald, Felix-Hausdorff-Street 3, 17489 Greifswald, Germany; 2 Diurnal Limited, Cardiff Medicentre, Heath Park, Cardiff, CF14 4UJ UK

**Keywords:** biorelevant dissolution, gastric media, liquids, quality assessment, soft foods

## Abstract

**Purpose:**

The objective of the present work was to screen whether a novel pediatric hydrocortisone granule formulation can be co-administered with common food matrices and liquids.

**Methods:**

Pediatric hydrocortisone granules were studied using a biopredictive *in vitro* approach. Experiments included an *in situ* chemical compatibility study of active ingredient and drug product with liquid dosing vehicles and soft foods commonly ingested by infants, pre-school- and school children. Drug solubility and stability experiments in the different vehicle types and, drug release/dissolution experiments mimicking age-related pediatric gastric conditions after administering the hydrocortisone granules together with the dosing vehicles and after different exposure/mixing times were performed.

**Results:**

In the simulated dosing scenarios applied in dissolution experiments, *in vitro* dissolution in gastric conditions was rapid and complete. Results of the chemical compatibility/stability studies indicated that mixing with the different dosing vehicles studied should not be an issue regarding drug degradation products.

**Conclusions:**

A novel *in vitro* approach ensuring a proper risk assessment of the use of dosing vehicles in the administration of pediatric dosage forms was established and applied to a novel pediatric hydrocortisone drug product. The studied dosing vehicles were shown to not alter performance of the drug product and are thus considered suitable for administration with hydrocortisone granules.

**Figure:**
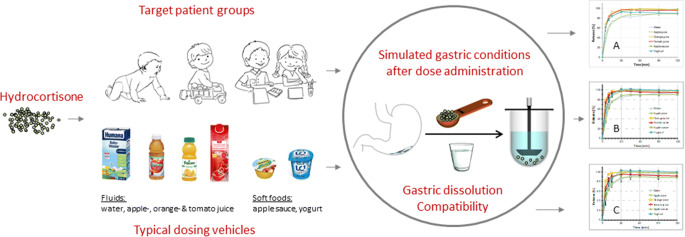
Graphical abstract

**Electronic supplementary material:**

The online version of this article (10.1007/s11095-020-02912-x) contains supplementary material, which is available to authorized users.

## Introduction

Adrenal insufficiency (AI) is an endocrine disorder caused by impaired synthesis and release of the essential hormone cortisol and can be classified into primary or secondary AI. Primary AI is caused by destruction or dysfunction of the adrenal cortex and presents with impaired cortisol and aldosterone production. Secondary AI is caused by a lack of pituitary adrenocorticotropic hormone (ACTH) which prevents the body from producing enough cortisol (1).

AI remains a significant cause of morbidity and mortality in children with 1 in 200 episodes of adrenal crisis resulting in death (2). In most cases pediatric AI is caused by the rare genetic condition congenital adrenal hyperplasia. Occasionally, it can be caused by autoimmune disease (Addison’s disease) or pituitary conditions. AI is a life-long and life-threatening condition and is associated with a multitude of symptoms that are often unspecific and thus make diagnosis difficult (1).

Patients diagnosed with AI require replacement therapy with glucocorticoids (steroids). To avoid risks of hypoglycemia and adrenal crisis associated with undertreatment, and to avoid growth suppression and reduced final height potential associated with steroid overdosing, appropriate replacement doses of glucocorticoids are important to determine in both primary and secondary AI in children (3). Hydrocortisone (the synthetic version of cortisol) represents the glucocorticoid of choice for treating pediatric AI patients (1,4). It is administered orally in two to four divided doses per day, where the single doses should be adjusted to the individual need based on body surface area (1,4). Since AI can present at birth and usually requires life-long and patient-tailored oral glucocorticoid medication, oral dosage forms that ensure acceptability in children of different age groups are an important prerequisite for safe, reliable and effective glucocorticoid substitution. However, to date optimization of hydrocortisone replacement therapy in children has been challenging, since until recently, no licensed oral dose-appropriate pediatric formulation of hydrocortisone was available (5). Most children with adrenal insufficiency have until now been treated with individualized pharmacy-compounded adult medication. In very young children such as newborns and infants compounding demonstrably bears risks of inaccurate dosing, poor disease control and potential adrenal crisis (5,6). Therefore, there is a strong need for licensed oral pediatric formulations approved by regulatory authorities such as the European Medicines Agency (EMA) and the United States (US) Food and Drug Administration (FDA) in the treatment of pediatric AI.

Hydrocortisone is a well-established glucocorticoid listed in the current US Pharmacopoeia (USP) and European Pharmacopoeia (Ph.Eur.). It is a neutral compound with acid-base properties (pka = 12.59) and lipophilicity (logP = 1.61) (7). In the adult setting, hydrocortisone has been reported to be a class II (high permeability and low solubility) compound (8) according to the biopharmaceutics classification system (BCS) (9). However, in the oral pediatric dose range hydrocortisone can be assigned to class I (high permeability and high solubility) where the highest marketed dose demonstrates pH-independent solubility in aqueous media across the physiologic pH range along the upper human gastro-intestinal (GI) tract (data on file). It should be noted that the current BCS refers to adult administration and that a pediatric BCS has not yet been established (10). For the physiological pH range of 1.2 to 6.8 an aqueous solubility of 0.35–0.40 mg/mL is reported (8,11). Thus, when considering an oral single dose of 5 mg in pediatric use, the dose:solubility ratio would be in the range of ~12.5–15 ml indicating that there should be no issues with regard to *in vivo* solubility, since even in very young children this is a reasonable fluid volume available in the upper GI tract (12). Alkindi^®^ (Diurnal Europe B.V., the Netherlands) (development program name Infacort^®^ and in the rest of the manuscript referred to as hydrocortisone granules) is an oral multiparticulate formulation of hydrocortisone that is available in unit doses of 0.5 mg, 1 mg, 2 mg and 5 mg, that was developed for use in children from birth as an oral replacement therapy in AI. The drug product is an immediate release (IR) dosage consisting of granules that are coated with taste-masking excipients to eliminate the bitter taste of hydrocortisone to ensure acceptability in children of all age categories. The granules are contained in size 00el hypromellose hard capsules which are not intended to be administered to the children but used as a carrier.

The hydrocortisone granules are intended to be dosed to pediatric patients by sprinkling the capsule content onto the child’s tongue. Alternatively, they can be placed on a dry spoon and administered into the patient’s mouth. Immediately after administration, the granules should be washed down with fluid, preferably drinking water, or for younger patients, breast or artificial milk (11). However, particularly when developing oral formulations for pediatric use, besides safety and efficacy, acceptability of the drug product is an important aspect to consider (13). To increase acceptability mixing with food and beverages is a common practice when otherwise the medication would not be accepted by the child. Consequently, typical instructions that can be found in the product information leaflet of marketed formulations for pediatric use often refer to co-administration with semi-solid (soft) food matrices or liquids. To ease the administration of the hydrocortisone granules, it would be advantageous to also provide the option to administer the capsule contents with different types of soft foods or fluids. However, this would require sufficient *in situ* stability of the drug (product) in these dosing vehicles to cover the expected administration timeframe. Moreover, the type and amount of food/fluid co-administered with the dosage form should not present with a relevant food effect, i.e. not interfere with *in vivo* drug release.

According to a recently published FDA draft guidance on the “Use of Liquids and/or Soft Foods as Vehicles for Drug Administration” (14) only those liquids and/or soft foods demonstrated to have no appreciable effect on drug product performance should be proposed as a dosing vehicle. Consequently, when the aim is to propose co-administration of the hydrocortisone granules, a study proving that the proposed dosing vehicles will not affect hydrocortisone stability and *in vivo* drug release is of upmost importance. The recent FDA guidance on information to be contained in pediatric labeling (15) does not include any details on the nature of tests to be conducted. Theoretically, compatibility and *in vivo* drug release could be assessed in an *in vivo* pharmacokinetic (PK) study. However, due to ethical concerns, it is impossible to perform such studies in children (16). The FDA guidance on exposure-response relationships (17) includes a “Pediatric Study Decision Tree” which justifies extrapolation from adult data into pediatric populations in cases where the course of the disease and effect of the drug are sufficiently similar in adults and pediatric patients. However, it is important to note that this extrapolation refers only to efficacy, but not to safety or dose adjustments. When adult data have been used to predict performance in pediatric populations there are examples of unexplained and sometimes adverse events (18–21).

A properly designed *in vitro* approach that addresses both the variability in composition and properties of different vehicles and pediatric GI physiology would be a valuable tool for assessing the compatibility and stability of pediatric drug products and common dosing vehicles.

In a previous study in Europe, under the agreed EMA Paediatric Investigation Plan (PIP – EMEA 001283-PIP01–12) the compatibility of the hydrocortisone granules with artificial milk, whole milk and breast milk was assessed. Results from this study indicated that compared to administration with drinking water, *in vivo* hydrocortisone exposure in neonates, infants and pre-school children is unlikely to be affected by co-administration with milk and the composition of milk co-administered with the hydrocortisone granules (11). To ensure the safety and efficacy of the hydrocortisone granules after co-administration with soft food and drink matrices commonly used to assist in the administration of pediatric medicines, the FDA requested proof of compatibility with soft food matrices and administration fluids that are commonly used in the US. The objective of the present work was thus to establish a biopredictive *in vitro* approach for studying i) the *in situ* chemical compatibility of the hydrocortisone granules with different dosing vehicles, ii) drug solubility and stability in the different vehicle types and iii) drug release/dissolution following a simulated co-administration of the hydrocortisone granules with these dosing vehicles after different exposure/mixing times.

## Materials and Methods

### Materials

Hydrocortisone granules in unit hydrocortisone doses of 0.5 mg, 1 mg, 2 mg or 5 mg, all containing the microparticulate batches # 0627/2014-P and # 0628/2014-P (both made from manufacturer batch # 1-984-00936) and hydrocortisone standard material, batch # W005614 (manufacturer batch # 1-983-61052) were obtained from Glatt Pharmaceutical Services GmbH & Co. KG, Binzen, Germany. The granules consist of an inert cellulose core that is covered by a hydrocortisone layer followed by a hypromellose sealing layer and an outer taste masking layer containing ethylcellulose and hypromellose to permit compliant oral dosing. Both sealing layer and taste-masking layer contain a small amount of magnesium stearate as a processing aid.

The analytical standards used for the quantification of hydrocortisone and its degradation products were all obtained from LGC Limited (Teddington, Middlesex, UK) and are given in Table I. All other chemicals and solvents were of analytical or gradient grade and purchased commercially.

**Table I:** Analytical Standards Used for the Quantification of Hydrocortisone and its Degradation Products

Compound	Batch #
Hydrocortisone	8.1
Cortisone	5980
Reichstein‘s Substance S	40844
Hydrocortisone acetate	9.1
Prednisolone	23623
Hydrocortisone for peak identification	2.0

### ***In Vitro*** Study Design

#### Dosage Strengths Tested

The proposed hydrocortisone doses per unit are 0.5 mg, 1 mg, 2 mg or 5 mg. Since these doses differ only in the number of multiparticulates contained in the capsule, it was regarded appropriate to focus on the most typical doses to be administered to pediatric patients in order to obtain an estimate of the *in vivo* performance of the drug product. Therefore, 2.5 mg or 5 mg representing typical doses administered to infants, pre-school children or school children, respectively, were subject of the study.

#### Age Groups to Be Addressed

The hydrocortisone granules are intended to be administered to children from birth. In a previous study, typical dosing conditions, i.e. co-administration of the granules with water, breast milk and formula milk for children pre-weaning have been studied (11). This study focused on age-groups that are typically weaned, i.e. infants, pre-school- and school children were simulated.

#### Foods Selected for the Compatibility Study

Compatibility with the common dosing vehicles including yogurt, apple sauce, orange juice, apple juice, tomato juice and water was studied. These vehicles were selected in keeping with the FDA guidelines. Since different brands of soft foods and fluids can differ in their composition and physicochemical properties, it was regarded important to consider such potential differences in the study design. Consequently, whereas it was considered appropriate to use a single source for water, for each of the other soft foods and fluids three different brands/qualities with likely different physicochemical characteristics were used. Wherever possible, one of these brands was from the US market. All fluids and soft foods selected for the compatibility study are given in Table II.

**Table II:** Fluid and Food Types and Sources

Fluid / Soft food	Commercial Product	Manufacturer / Source	Batch #
Water	Humana Babywasser	Humana GmbH, Herford, Germany	47159351
Apple juice	Tropicana apple juice	Tropicana Manufacturing Company Inc., Bradenton, USA	32950086112
	Alosa Apfelsaft klar	Brands & Systems BSG GmbH, Hamburg, Germany	1124H
	Albi Apfelsaft klar	Albi GmbH, Berghuelen, Germany	L251113 M3B2
Orange juice	Tropicana orange juice(with pulp)	Tropicana Manufacturing Company Inc., Bradenton, USA	ZMZ K10:34
	Hohes C Orangensaft(without pulp)	Eckes-Granini Deutschland GmbH, Nieder-Olm, Germany	300 & 329
	Amecke Sanfte Saefte, Orangensaft (without pulp)	Amecke Fruchtsaft GmbH, Menden, Germany	03062SN15030
Tomato Juice	Rewe Beste Wahl Tomatensaft	Rewe Markt GmbH, Koeln, Germany	L2E2
	Tomatensaft von Sonnlaender	Sonnlaender Getraenke GmbH, Rostock, Germany	191015
	Alnatura Tomatensaft	Alnatura GmbH, Bickenbach, Germany	61049
Apple sauce	MOTT’S applesauce natural	Mott’s Inc., Plano, USA	111715WA
	Babylove Apfel pur	dm-drogerie markt GmbH + Co. KG, Karlsruhe, Germany	LS272
	Oberlausitzer Apfelmus	Lausitzer Fruechteverarbeitung GmbH, Sohland, Germany	L14 16:05 LK
Yogurt	LC1 Pur Nestlé	Lactalis Nestlé Frischprodukte Deutschland GmbH, Kehl/Rhein, Germany	12/02H & 28/05 K
	Alpro Sojajoghurt	Alpro C.V.A., Wevelgem, Belgium	Y021709:16 1
	Weihenstephan Frischer Joghurt mild, 3.5% Fett	Molkerei Weihenstephan GmbH & Co. KG, Weihenstephan, Germany	BY718.150504:4902

#### Physicochemical Characterization of the Dosing Vehicles

Physicochemical characterization of all fluids and soft foods presented in Table II included the following parameters: pH value and buffer capacity, osmolality, surface tension and viscosity. Except for osmolality parameters were recorded at two temperatures, i.e. 25°C and 37°C as described in (22). Unless otherwise indicated, all experiments were run in sextuplicate and results expressed as mean ± S.D. Measurements were performed as follows:

The pH value was measured with a pH-meter (Five Easy Plus, Metter Toledo GmbH, Giessen, Germany). Using the same pH-meter for pH control, the buffer capacity was quantified by potentiometric titration with 0.1 M or 0.2 M hydrochloric acid, respectively.

Osmolality was measured via the freezing-point depression method using a semi-micro osmometer (K-7400, Knauer, Berlin, Germany). Most of the fluids and foods required dilution prior to the measurement. Thus, for these soft foods a set of appropriate dilutions was prepared with demineralized water. These dilutions were then first mixed for 1 min using a Vortex mixer (VWR Reagenzglasschuettler, VWR International GmbH, Darmstadt, Germany) and subsequently centrifuged for 15 min at 4000 rpm (Eppendorf Centrifuge 5702 R, Eppendorf AG, Hamburg, Germany). After centrifugation, the aqueous phase of the diluted foods was used to measure the osmolality. A linear relationship between food concentration and osmolality was observed (R^2^ > 0.995) for the entire set of dilutions. Consequently, it was possible to extrapolate to the osmolality of the undiluted soft foods. Osmolality of some of the soft foods could not be assessed by the freezing-point depression method. For these media, i.e. MOTT’s and Babylove apple sauce and Alpro yogurt, osmolality was extrapolated by preparing and measuring of a set of appropriate dilutions with a vapor pressure osmometer (No. 11.00, Knauer, Berlin, Germany).

The surface tension was determined with a ring tensiometer (K11, Kruess GmbH, Hamburg, Germany). As experienced in the osmolality measurements, the surface tension of apple sauce and yogurt could not be directly assessed. Therefore, again a set of appropriate dilutions was prepared. Both at 25°C and 37°C no major change in surface activity could be observed when comparing surface tension of the different dilutions. This indicates that the surfactant concentrations were above the critical micelle concentration (CMC). The surface tension of the undiluted soft foods was thus estimated from the mean (*n* = 24 ± S.D. for MOTT’s apple sauce, *n* = 36 for Babylove apple sauce and *n* = 30 for Alpro yogurt) surface tension of the respective sets of dilution.

Due to the very different consistencies of the samples, it was necessary to use two methods for the investigation of viscosity. The viscosity of all Newtonian fluids was determined with two different Ubbelohde viscometers (type I, K = 0.01008 mm^2^/s^2^ and type II, K = 0.09939 mm^2^/s^2^, both from LaborTherm, Jena, Germany). As expected, some of the fluids and the soft foods did not exhibit Newtonian flow characteristics. The rheological profiles of these samples, i.e. orange juice with pulp, tomato juice, apple sauce, and yogurt were obtained by measuring shear stress over a range of shear rates. Rheological profiles were recorded with rotational viscometers operating according to the Searle principle (cup and bob). These were either a proRheo R180 apparatus (proRheo GmbH, Althengstett, Germany) equipped with cup size 2 (22) or a Brookfield DV3T apparatus (Brookfield, Middleborough, MA, USA) equipped with the ULA-DIN-6Y cup and the DIN 87 spindle or the DAA-1 cup and the DIN 85 spindle, respectively.

Besides the physicochemical characterization, fluids and soft foods were also screened for energy and nutrient composition.

#### Dissolution and Solubility Study

##### Dissolution Media

Dissolution media were chosen to simulate gastric conditions immediately after dose administration. Therefore, the dissolution media applied were mixtures of physiological fasted resting volumes of simulated gastric fluids with age-appropriate pH, a typical amount of soft food or fluid co-administered with a single dose (1 teaspoon) and an age-appropriate volume of fluid that is anticipated to be ingested after administration. One brand of each soft food/fluid product, preferably a product from the US market, was selected for the dissolution studies. The products that were applied in the dissolution studies were Humana Babywasser, Tropicana apple juice, Tropicana orange juice, Rewe Beste Wahl tomato juice, MOTT’S applesauce natural and LC1 Pur Nestlé yogurt. Table III gives a detailed overview of the different dosing scenarios mimicked in the dissolution experiments.

**Table III:** Estimated Gastric Content in Children of Different Age Categories After Co-administration of a Typical Hydrocortisone Dose

Age group	Type of soft food / fluid	Test dose	Amount of co-aministered food/fluid	Resting gastric volume	Resting gastric pH range	Volume of co-administered water	Total gastric volume	
Infants & pre-school children	Water	2.5 mg	1 teaspoon = 5 ml	10 ml	pH 1.8–4.0	35 ml	50 ml	
	Apple juice							
	Orange juice							
	Tomato juice					85 ml	100 ml	
	Apple sauce							
	Yogurt							
School children	Water	5.0 mg	1 teaspoon = 5 ml	25ml	pH 1.8	170 ml	200 ml	
	Apple juice							
	Orange juice							
	Tomato juice							
	Apple sauce							
	Yogurt							

##### Dissolution Test Setup and Sampling Procedure

**Fig. 1:**
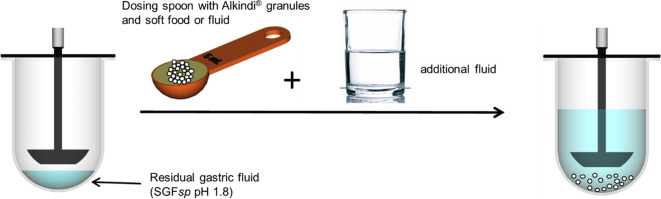
General test design for the dissolution experiments.

Most of the typical gastric content volumes to be mimicked in the dissolution study did not allow the use of standardized dissolution equipment. Thus, non-pharmacopoeial test equipment would have been required for performing the experiments. However, since the objective was to run the experiments under standardized test conditions and to make the results comparable to those from the first part of the study (11), experiments were performed with the Mini-Paddle apparatus using a media volume of 200 or 195 ml, respectively. Where necessary, i.e. when mimicking gastric conditions in infants and pre-school children, hydrocortisone dose, vehicle- and additional fluid volumes were up-scaled proportionally. For infants and pre-school children scaling factors of 4 or 2 were applied when mimicking co-administration of 35 ml (setup 1) or 85 ml water (setup 2), respectively. The test design for school children did not require the use of a scaling factor. Experiments in the Mini-Paddle apparatus were performed as follows: simulated residual gastric fluid, i.e. Simulated Gastric Fluid without pepsin (SGF*sp*) pH 1.8 was placed into the vessel and preheated to 37°C. Then, at room temperature the dosing vehicle was placed in a graduated dosing spoon and the hydrocortisone granule test dose was mixed with the vehicle (sprinkling and stirring with a small plastic rod for 5 s). The resulting mixture was immediately added to the preheated gastric medium. For the experiments with soft food an additional set of experiments was performed where the soft food:granule mixture was kept at room temperature for 60 min before simulating administration/ingestion (worst case scenario assumed for a child refusing to take the medicine at the time of intended administration). When mimicking hydrocortisone granule co-administration with soft foods, water of 37°C was added to the vessel to simulate administration of some extra fluid after dosage form administration. In those cases where co-administration of the dosage form with liquids (apple juice, orange juice and tomato juice) was mimicked, we assumed that the child would drink the same type of liquid after administration and thus, instead of water, added the corresponding volumes of the respective fluid types at 37°C. The test setup is shown in Fig. 1. Over the duration of the dissolution experiment the media temperature was maintained at 37.0 ± 0.5°C and the paddle speed was set at 100 rpm (23,24). The total time of the dissolution experiments was 120 min. Samples of 3 ml were removed at predetermined time points, i.e. 5, 10, 15, 30, 45, 60, 90, and 120 min. The sample volume was replaced by fresh medium, i.e. a 1:4 mixture of SGF*sp* pH 1.8, respectively and water in all experiments. All experiments were run in triplicate.

##### Solubility Experiments

In addition to the dissolution experiments a small series of hydrocortisone solubility experiments was performed in selected dissolution media. The objective of these experiments was to estimate the solubility of hydrocortisone in a mixture of residual gastric fluid, vehicle and co-administered fluid. Results of the solubility experiments should be helpful in assessing the dissolution results, particularly with respect to sink conditions. As it was not possible to perform experiments in all mixtures used in the dissolution experiments, we decided to select the infant test scenario assuming an infant/pre-school child test scenario with a residual gastric fluid volume of 10 ml and a pH of 1.8 receiving the medication with a teaspoon (5 ml) of fluid or soft food and drinking 85 ml of water or the fluid used for dose administration immediately after dosing. Consequently, the test media composition for the solubility experiments was SGF*sp*:fluid 10%:90% for apple juice, orange juice and tomato juice and SGF*sp*:soft food:water 10%:5%:85% for apple sauce and yogurt. All solubility experiments were performed in triplicate using the shake-flask method at 37.0 ± 0.5°C. Experiments were performed as follows: An excess of drug was added to a 5 ml flask containing 4 ml of each individual test medium and the resulting suspension was stirred with a magnetic stirring bar at 220 rpm. During the mixing period, the flasks were inspected to ensure that each flask contained undissolved, excess drug. Drug was added as necessary to maintain an excess. Samples were taken 24 h after the start of the experiment, i.e. when an equilibrium concentration of the compound in the test medium was reached.

##### Sample Preparation for High-Performance Liquid Chromatography (HPLC) Analysis

Aqueous samples from dissolution and solubility testing in baby water were filtered via a 0.22 μm polyvinylidene fluoride (PVDF) filter (diameter 25 mm, Millex-HV, Merck Millipore, Darmstadt, Germany) immediately after sampling. To adapt the sample composition to that of the mobile phase of (80% water and 20% tetrahydrofuran (THF) v/v) used for HPLC analysis, 0.8 ml of each sample was added to 0.2 ml THF. After homogenization, the resulting mixtures were analyzed by HPLC.

Samples from dissolution and solubility experiments mimicking co-administration with apple juice were first filtered through a 0.22 μm PVDF filter, then 800 μl of the filtrate were added into a 1.5 ml polypropylene SafeSeal tube (Sarstedt, Nuembrecht, Germany) filled with 200 μl of THF. The resulting mixtures were then centrifuged for 30 min at a speed of 13,000 rpm. Finally, the supernatant was filtered through a 0.45 μm PVDF filter (diameter 13 mm, Whatman Schleicher & Schuell, Dassel, Germany) and transferred into HPLC vials, as necessary diluted with mobile phase and analyzed by HPLC.

Samples from dissolution and solubility testing simulating co-administration with orange juice, tomato juice and yogurt were first transferred into 1.5 ml polypropylene SafeSeal tubes (Sarstedt, Nuembrecht, Germany) and centrifuged for 1 min. The supernatant was then transferred into a new tube and again centrifuged for 30 min at a speed of 13,000 rpm resulting in phase separation. In the next step, 1 ml of the aqueous phase was removed and transferred into a new cap. Subsequently, 0.5 ml of acetonitrile was added, and the samples were again centrifuged as described before. In the last step 800 μl of the supernatant were removed and added to a new tube containing 200 μl THF. The resulting mixtures were vortexed and then filtered via a 0.45 μm PVDF filter and transferred into HPLC vials, as necessary diluted with mobile phase and analyzed by HPLC.

Samples from dissolution and solubility testing simulating co-administration with apple sauce were first filtered through a 0.22 μm PVDF filter and then transferred into 1.5 ml polypropylene SafeSeal tubes (Sarstedt, Nuembrecht, Germany). They were then centrifuged for 30 min at a speed of 13,000 rpm resulting in phase separation. In the next step, 1 ml of the aqueous phase was removed and transferred into a new cap. Subsequently, 0.5 ml of acetonitrile was added, and the samples were again centrifuged as described before. In the last step 800 μL of the supernatant were removed and added to a new tube containing 200 μL THF. The resulting mixtures were vortexed and then filtered via a 0.45 μm PVDF filter and transferred into HPLC vials, as necessary diluted with mobile phase and analyzed by HPLC.

To ensure that, due to these various separation and dilution steps all the drug that had dissolved in the aqueous phase prior to analysis could be properly quantified, for each of the experiments, the same separation and dilution steps were performed using the respective media containing known amounts of dissolved hydrocortisone. For this purpose, the amount of drug that would freely dissolve in the respective medium was calculated based on results of the solubility experiments. The recovery for these so-called standard samples was then calculated. The concentration of hydrocortisone released/dissolved in the individual test media in the dissolution experiments was then corrected by multiplication with the recovery factor obtained for the standard samples. The individual correction factors applied in the study are given in Table IV.

**Table IV:** Correction Factors Used for Calculating Drug Concentration in the different liquid and semi-solid dosing vehicles

Medium	Correction factor
Apple juice	1.003
Orange juice	1.014
Tomato juice	0.992
Apple sauce	1.037
Yogurt	1.019

##### Sample Analysis

After appropriate sample preparation, samples were analyzed by HPLC (Waters system consisting of an 2707 autosampler, a 1525 binary pump, a 2998 photodiode array detector and the Breeze 2 software, Waters GmbH, Eschborn, Germany) using a Waters Symmetry C18, 3.5 μm, 75 mm × 4.6 mm column, equipped with a Waters Symmetry C18 Sensity Guard, 5 μm, 20 mm × 3.9 mm precolumn (both from Waters GmbH, Eschborn, Germany), both equilibrated at 45°C, under isocratic conditions using a tetrahydrofuran (THF)/water (20:80 v/v) mobile phase at a flow rate of 1.5 ml/min. Hydrocortisone was detected with ultraviolet (UV) detection at 254 nm. This method had been adapted from Glatt Pharmaceutical Services GmbH & Co. KG and was partly re-validated before use: The linearity was screened for concentration ranges of 0.001–0.050 mg/ml and 0.01–0.40 mg/ml, respectively, in both cases R^2^ was 1.000. Moreover, with dilution sets in the same concentration ranges, the accuracy of the mean as well as the precision was checked. Both parameters were in the limits of ± 5%. Therefore, the method was regarded as appropriate for the intended use. The injection volume was 30 μl for samples from the solubility experiments and 15–30 μl for those from the dissolution experiments.

#### Chemical Compatibility/Stability Study

The *in situ* chemical stability profile, i.e. the compatibility of the hydrocortisone granules with soft food matrices and fluids used for mixing and administration was tested over the chosen time period to simulate a range of administration- and clinical practice scenarios. Beside chemical stability of the active pharmaceutical ingredient (API, i.e. hydrocortisone), the visual appearance and the pH of the vehicle was assessed. Experiments were performed as follows:

The contents of two capsules each containing granules with 5 mg of hydrocortisone (Glatt Pharmaceutical Services GmbH & Co. KG, batch # 0628/2014, material no. 1-984-00936) were mixed with 10 ml (corresponding to a 5 mg dose administered on a teaspoon of 5 ml) of each of the vehicles. Two different dosing scenarios simulating immediate and delayed administration after mixing were addressed. Consequently, two separate sample sets with granule resting times of 5 min and 60 min in the different vehicles were prepared. To assess the impact of the granule formulation on API stability, a corresponding set of experiments was conducted with hydrocortisone standard substance (1 mg hydrocortisone + 10 ml vehicle) (Glatt Pharmaceutical Services GmbH & Co. KG, batch # W005614, material no. 1–983-01052) for comparison. All experiments were performed in triplicate and at room temperature (22–25°C). Prior to the experiments the content of hydrocortisone of the examined capsules had been determined as 100.2 ± 0.05% (mean of *n*=3 ± S.D.).

With regard to the detailed experimental procedures to be applied the six vehicles were divided into two groups, liquid vehicles (water, apple juice, orange juice and tomato juice) and semi-solid vehicles (apple sauce and yogurt).

##### Chemical Compatibility with Liquid Vehicles – Hydrocortisone Granules

The amount of hydrocortisone granules corresponding to a dose of 10 mg of hydrocortisone was added to a 20 ml glass vial. Then 10 ml of the respective vehicle, equilibrated to room temperature, were added into the vial and the mixtures were left standing for 5 or 60 min, respectively. Temperature and pH of all vehicles were measured before the experiment and at the end of the resting period. Samples of the vehicle matrix were taken after 5 or 60 min and immediately prepared for HPLC analysis. Due to the complexity of the vehicle compositions, vehicle-specific sample preparation methods had to be applied:

Water: Samples of 2 ml were removed, transferred into a 2 ml polypropylene SafeSeal tube (Sarstedt, Nuembrecht, Germany) and centrifuged for 1 min with a speed of 13,000 rpm to separate the vehicle matrix from the granules removed with the vehicle sample. In the next step the supernatant was filtered via a 0.45 μm PVDF filter (diameter 13 mm, Whatman Schleicher & Schuell, Dassel, Germany) into a test tube. To adapt the sample composition to the initial mobile phase composition of the media gradient used for HPLC (76% water and 24% acetonitrile v/v) and also to stop further potential degradation of hydrocortisone after sampling, 0.2 ml acetonitrile was added to 0.8 ml of the sample. Subsequently, samples were analyzed by HPLC.

Apple juice, orange juice, tomato juice: Samples of 2 ml were removed, transferred into a 2 ml polypropylene SafeSeal tube and centrifuged for 1 min with a speed of 13,000 rpm. Subsequently, 1.6 ml of the supernatant was transferred into another tube and 0.4 ml of acetonitrile was added to stop further potential degradation of hydrocortisone. The resulting mixture was then centrifuged for 30 min at 13,000 rpm. Subsequently, 0.2 ml of the supernatant was transferred into a new cap, 1.8 ml acetonitrile was added, and the samples were again centrifuged as described before. In the last step, 0.5 ml of the supernatant was transferred into a new tube and 1.5 ml of water of HPLC quality was added to adapt the sample composition to the initial mobile phase composition of the media gradient used for HPLC. The resulting mixture was filtered (0.45 μm PVDF filter) into an HPLC vial and analyzed by HPLC.

##### Chemical Compatibility with Liquid Vehicles – Hydrocortisone Standard

A 20 ml glass vial equipped with a 8 mm polytetrafluoroethylene (PTFE) coated magnetic stirring bar and containing 0.4 mg of hydrocortisone reference substance was placed on a magnetic stirring plate (IKA RT 15 P, IKA-Werke GmbH & Co. KG, Germany) Then, 4 ml of the respective vehicle, equilibrated to room temperature, were added. The mixture was agitated with the magnetic stir bar and temperature and pH of all vehicles were measured before the experiment and at the end of the stirring period. Samples of the vehicle matrix were removed after 5 or 60 min and immediately prepared for HPLC analysis. Again, due to the complexity of the vehicle compositions, vehicle-specific sample preparation methods had to be applied:

Water: Samples of 2 ml were removed with a glass syringe and filtered into a test tube via a 0.45 μm PVDF filter. To adapt the sample composition to the initial mobile phase composition of the media gradient used for HPLC (76% water and 24% acetonitrile v/v) and also to stop further potential degradation of hydrocortisone after sampling, 0.2 ml acetonitrile was added to 0.8 ml of the sample and the samples were analyzed by HPLC.

Apple juice, orange juice, tomato juice: Samples of 1.6 ml were removed, transferred into a 2 ml polypropylene tube and 0.4 ml of acetonitrile was added. The resulting mixture was then centrifuged for 30 min at 13,000 rpm. Following centrifugation, 0.2 ml of the supernatant was transferred into a new cap, 1.8 ml acetonitrile were added, and the samples were again centrifuged as described before. Finally, 0.5 ml of the supernatant was transferred into a new tube and 1.5 ml of water of HPLC quality was added. The resulting mixture was filtered (0.45 μm PVDF filter) into an HPLC vial and analyzed by HPLC.

##### Chemical Compatibility with Semi-Solid Vehicles – Hydrocortisone Granules

Due to the composition and texture of apple sauce and yogurt that did not provide direct access to an aqueous phase that could be sampled and also confronted the analyst with the task to take samples from a mixture of freely dispersed fine granules in a semi-solid vehicle, the experimental test setup was adapted as follows: at room temperature 8.84 g apple sauce (equivalent to 10 ml) or 8.64 g yogurt (equivalent to 10 ml) were spread onto the surface of a porcelain frit (pore size 2) of a Buchner (vacuum suction) funnel. Then, the amount of hydrocortisone granules corresponding to a dose of 10 mg of hydrocortisone was sprinkled on the surface of the vehicle and incorporated by gentle and brief stirring with a plastic spoon. The Buchner funnel was then placed onto a suction flask connected to a vacuum pump. After 5 or 60 min, respectively, vacuum was applied, and the filtered sample was collected into a test tube inside the suction flask. Temperature and pH value of all vehicles were measured before the experiment and at the end of the resting period. Following filtration samples were immediately prepared for HPLC analysis as follows: 0.8 ml of the sample was transferred into a 2 ml polypropylene SafeSeal tube, 0.2 ml of acetonitrile was added, and the mixture was centrifuged for 30 min at 13,000 rpm. Then, 0.2 ml of the supernatant was transferred into a new tube, 1.8 ml acetonitrile was added, and the samples were again centrifuged as described before. Finally, 0.5 ml of the supernatant was transferred into a new tube, 1.5 ml water of HPLC quality was added. The resulting mixture was filtered through a 0.45 μm PVDF filter into a HPLC vial and analyzed by HPLC.

##### Chemical Compatibility with Semi-Solid Vehicles – Hydrocortisone Standard

A 20 ml glass vial equipped with a 8 mm magnetic stirring bar (PTFE) and containing 0.4 mg of hydrocortisone reference substance was placed on a magnetic stirring plate (IKA RT 15 P, IKA-Werke GmbH & Co. KG, Germany). Then, 4 ml of the respective vehicle, equilibrated to room temperature, were added. The mixture was agitated with the magnetic stir bar. Temperature and pH value of all vehicles were measured before the experiment and at the end of the resting period. Samples of the vehicle matrix were taken after 5 or 60 min and immediately prepared for HPLC as follows: 1.6 ml of the sample was transferred into a 2 ml polypropylene SafeSeal tube, 0.4 ml of acetonitrile was added and the mixture was centrifuged for 30 min at 13,000 rpm. Then, 0.2 ml of the supernatant was transferred into a new tube, 1.8 ml acetonitrile was added, and the samples were again centrifuged as described before. Finally, 0.5 ml of the supernatant was transferred into a new tube, 1.5 ml water of HPLC quality was added. The resulting mixture was filtered through a 0.45 μm PVDF filter into a HPLC vial and analyzed by HPLC.

##### Sample Analysis

After appropriate sample preparation, samples were analyzed by HPLC (2707 autosampler, 1525 binary pump, 2998 photodiode array detector, Breeze 2 software, all Waters) using a LiChrospher 100 RP-18, 5 μm, 250 mm × 4.6 mm column, equipped with a LiChrospher 100 RP-18, 5 μm, 4 mm × 4 mm precolumn (both from Merck, Darmstadt, Germany) equilibrated at ambient temperature, under gradient conditions (see Table V). Water and acetonitrile were used as eluents and the flow rate was set at 0.6 ml/min. Hydrocortisone and the impurities/degradation products were detected with UV detection at 254 nm.

**Table V:** HPLC Gradient Applied in the Chemical Compatibility Analysis

Time [min]	Water [%]	Acetonitrile [%]
0	76	24
18	74	26
32	55	45
48	30	70
50	76	24
60	76	24

This HPLC method had been adapted from Glatt Pharmaceutical Services GmbH & Co. KG and was partly re-validated for hydrocortisone (batch W005614) before use: The linearity was screened for the concentration range of 0.0015–0.150 mg/ml with R^2^ > 0.9999. Moreover, with dilution sets in the same concentration range, the accuracy of the mean as well as the precision was checked. Both parameters were in the limits of ± 5%. Therefore, the method was regarded as appropriate for the intended use. The injection volume was 35 μl for samples from mixtures in water and apple juice and 95 μl for those in orange juice, tomato juice, apple sauce and yogurt. The following standards were used to screen samples for hydrocortisone content and the following related substances and impurities: Hydrocortisone, Cortisone – impurity B, Prednisolone – impurity A, Hydrocortisone Acetate – impurity C, Reichstein’s substance S - impurity F, Hydrocortisone for peak identification – contains impurities D, E, G, H, I and N. Chromatograms were evaluated according to the criteria for the determination and quantification of related substances given in the hydrocortisone monograph of the Ph.Eur.

## Results and Discussion

### Physicochemical Properties of the Dosing Vehicles

**Fig. 2:**
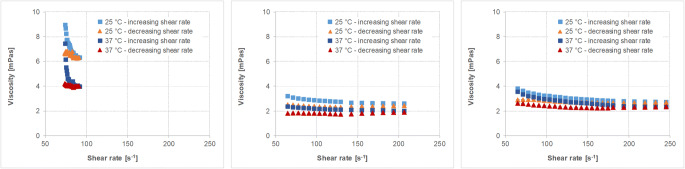
Viscosity profiles of different orange juice products, i.e. Tropicana (left), Hohes C (middle) and Amecke (right panel) at 25°C and 37°C and increasing and decreasing shear rates (mean of *n*=3, S.D. not shown).

**Fig. 3:**
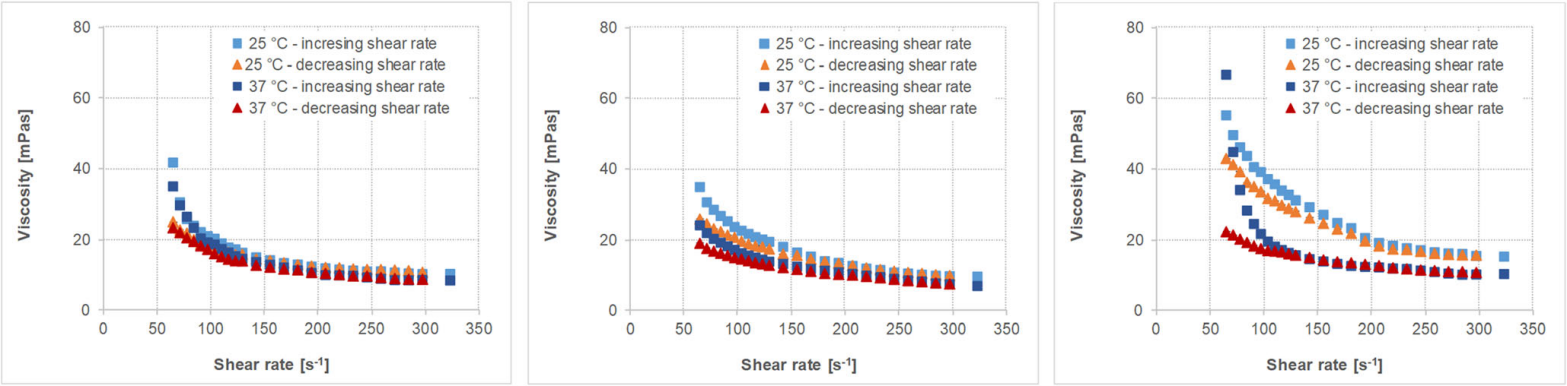
Viscosity profiles of different tomato juice products, i.e. Rewe (left), Sonnlaender (middle) and Alnatura (right panel) at 25°C and 37°C and increasing and decreasing shear rates (mean of *n*=3, S.D. not shown).

**Fig. 4:**
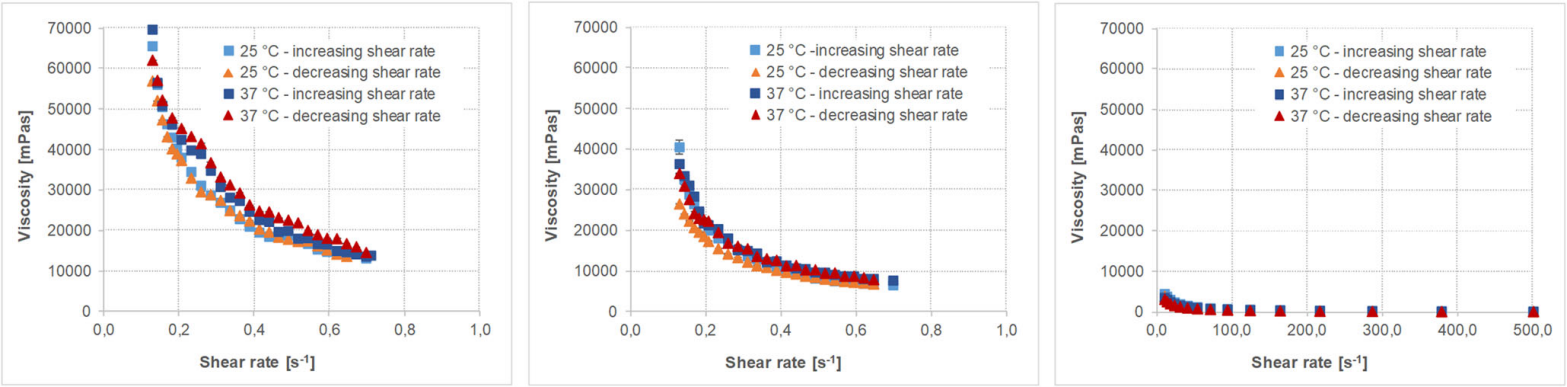
Viscosity profiles of different apple sauce products, i.e. MOTT’s (left), babylove (middle) and Lausitzer (adapted from data from Kersten E, Barry A, Klein S., Pharmazie, 2016, 71 (3):122–7 (16) (measured with a different cup and bob system in a different range)) (right panel) at 25°C and 37°C and increasing and decreasing shear rates (mean of *n*=3, S.D. not shown).

**Fig. 5:**
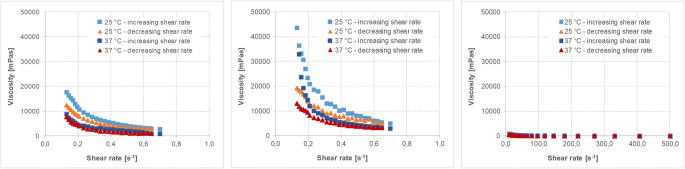
Viscosity profiles of different yogurt products, i.e. LC 1 Nestlé (left), Alpro (middle) and Weihenstephan (adapted from data from Kersten E, Barry A, Klein S., Pharmazie, 2016, 71 (3):122–7 (16) (measured with a different cup and bob system in a different range)) (right panel) at 25°C and 37°C and increasing and decreasing shear rates (mean of *n*=3, S.D. not shown).

The physicochemical characteristics of the different fluids and soft foods are given in Table VI. The viscosity profiles of the vehicles with non-Newtonian flow behavior are shown in Figs. 2, 3, 4, 5.

**Table VI:** Mean Values (± S.D.) of the Different Physicochemical Parameters of Fluids and Soft Foods (*n*=6 per measurement)

		Fluids										Soft foods					
		Water	Apple juice			Orange juice			Tomato juice			Apple sauce			Yogurt		
Parameter	T	Humana	Tropicana	Alosa	Albi^##^	Tropicana	Hohes C	Amecke ^##^	Rewe ^##^	Sonnlaender	Alnatura	MOTT’s	babylove	Lausitzer ^##^	LC 1 Nestlé	Alpro	Weihenstephan ^##^
pH-value	25°C	7.31 (0.01)	3.64 (0.00)	3.25 (0.01)	3.47 (0.03)	3.87 (0.00)	3.64 (0.01)	3.87 (0.04)	4.30 (0.01)	4.23 (0.02)	4.28 (0.01)	3.35 (0.01)	3.75 (0.01)	3.71 (0.02)	4.19 (0.00)	4.48 (0.01)	4.27 (0.02)
	37°C	7.89 (0.01)	3.64 (0.00)	3.24 (0.01)	3.49 (0.01)	3.87 (0.00)	3.66 (0.00)	3.85 (0.02)	4.27 (0.00)	4.21 (0.00)	4.27 (0.03)	3.35 (0.00)	3.74 (0.01)	3.70 (0.01)	4.17 (0.01)	4.45 (0.00)	4.26 (0.01)
Buffer capacity [mEq/pH/L]	25°C	0.11 (0.00)	23.4 (0.0)	31.1 (0.2)	33.4 (1.7)	49.4 (0.2)	52.2 (0.3)	48.4 (2.1)	52.6 (1.0)	41.9 (0.5)	39.1 (0.1)	32.2 (0.6)	28.1 (0.2)	26.3 (1.9)	98.3 (0.7)	72.7 (1.1)	93.8 (4.1)
	37°C	0.06 (0.0)	23.1 (0.2)	31.3 (0.3)	33.9 (0.3)	48.5 (0.3)	52.1 (0.3)	49.1 (1.1)	53.9 (0.2)	42.1 (0.2)	40.3 (0.7)	31.9 (0.4)	27.7 (0.3)	25.8 (0.4)	98.7 (0.7)	75.7 (1.6)	91.5 (0.3)
Osmolality [mOsmol/kg]		4 (1)	677 (9)	710 (10)	677 (5)	542 (22)	564 (20)	558 (8)	519 (8)	489 (4)	471 (6)	554^~^ (5)	706^~^ (5)	1052 (5)	488 (20)	245^~^ (2)	484 (6)
Surface tension [mN/m]	25°C	70.2 (0.36)	55.32 (1.94)	60.00 (0.65)	64.17 (0.32)	45.35 (1.51)	47.02 (1.33)	42.63 (2.10)	42.12 (0.25)	38.89 (0.99)	42.79 (2.62)	52.84 (3.65)	49.32 (5.02)	45.00 (0.33)	45.70 (0.90)	50.83 (0.70)	45.23 (0.21)
	37°C	68.74 (0.46)	53.87 (2.74)	60.82 (1.46)	62.51 (0.45)	43.50 (0.71)	45.54 (0.3)	49.10 (1.10)	39.42 (0.22)	37.86 (0.48)	42.82 (1.34)	49.46 (3.49)	45.08 (4.15)	42.27 (0.22)	44.42 (0.66)	47.86 (0.83)	43.82 (0.16)
Viscosity [mPa*s]	25°C	0.91 (0.00)	1.29 (0.00)	1.27 (0.01)	1.26 (0.00)	‡	‡	‡	‡	‡	‡	‡	‡	‡	‡	‡	‡
	37°C	0.72 (0.00	0.96 (0.00)	0.94 (0.01)	0.96 (0.00)	‡	‡	‡	‡	‡	‡	‡	‡	‡	‡	‡	‡

## data from Kersten E, Barry A, Klein S., Pharmazie, 2016, 71(3):122-7(16), ‡ measured with a rotational viscometer, ˜ measured with a vapor pressure osmometer

Results show that besides water, the pH values of all tested fluids are in a very narrow, slightly acidic pH range. Moreover, within the three selected fluids and foods of the same type (having similar nutrient properties), in most of the cases there is little variability in the physicochemical properties. Only the viscosity or the flow behavior, respectively, are quite different. Whereas water and apple juice show Newtonian flow behavior and have a rather low viscosity similar to water, the orange juices containing pulp, tomato juice and the soft foods are characterized by non-Newtonian flow behavior.

The rheological profiles of the three orange juices shown in Fig. 2 indicate that the viscosity of orange juice is affected by shear stress. However, the flow behavior is still close to that of Newtonian fluids and the viscosity of all orange juices is lower than 10 mPa*s. The somewhat strange appearance of the flow curves of Tropicana orange juice is most likely a result of the pulp which affects the results in a rather random manner.

Viscosity of the tomato juices (Fig. 3) is somewhat higher than that observed for all other fluids used in the study, but the rheological profiles of the three tomato juices screened are very similar.

Viscosity of the soft foods (Figs. 4 and 5) is higher than that of the fluids and much stronger affected by the applied shear rate. The vehicles as such might thus have an impact on drug diffusion and consequently drug release from a co-administered dosage form.

### Hydrocortisone Solubility in Different Simulated Gastric Conditions

Results from the solubility experiments (Table VII) were in the range of 0.1 mg/ml to 0.4 mg/ml hydrocortisone dissolved after 24 h. These results indicate that in the dissolution test conditions applied in the present study, 10-fold sink conditions will be provided in most of the media and that only when simulating co-administration with tomato juice followed by a small fluid volume in the infant/pre-school child test design not even 3-fold sink conditions might be achieved. However, when assessing these results, it should be noted that the objective of this initial set of solubility studies was to get a coarse estimate of the maximum amount of hydrocortisone that can dissolve in the gastric environment after co-administration with different dosing vehicles and some additional fluid. For this purpose, it was sufficient to remove just one sample from each individual glass vial at a time point where equilibrium solubility was expected to be reached.

**Table VII:** Solubility of Hydrocortisone in Different Test Media at 37°C (mean of *n*=3 ± S.D.) and the Corresponding Media pH at the Beginning and End of Each Experiment (*n*=1)

Medium	Solubility - 24 h c [mg/ml]	S.D.	pH - start	pH - 24 h
SGF*sp* pH 1.8 – Apple juice (10%/90%)	0.331	0.026	3.61	3.54
SGF*sp* pH 1.8 – Orange juice (10%/90%)	0.281	0.086	3.88	3.75
SGF*sp* pH 1.8 – Tomato juice (10%/90%)	0.101	0.071	4.12	4.06
SGF*sp* pH 1.8 – Apple sauce – Water (10%/5%/85%)	0.397	0.029	3.02	2.93
SGF*sp* pH 1.8 – Yogurt – Water (10%/5%/85%)	0.387	0.011	4.04	3.96

### Dissolution of Hydrocortisone Granules in Different Simulated Gastric Conditions

**Fig. 6:**
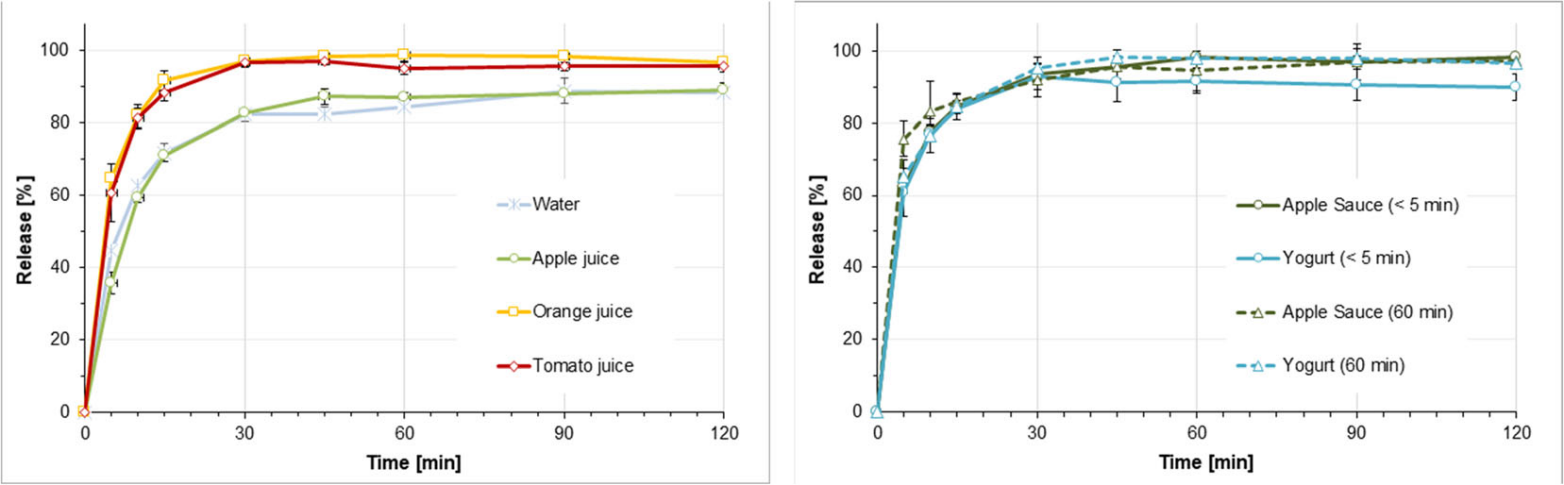
(**a**) Dissolution profiles obtained when simulating co-administration of granules containing 2.5 mg hydrocortisone with 1 teaspoon (5 ml) of fluid + 35 ml fluid in infants or pre-school children with a resting gastric fluid pH of 1.8, mean of *n*=3 ± S.D. **(b**) Dissolution profiles obtained when simulating co-administration of granules containing 2.5 mg hydrocortisone with 1 teaspoon (5 ml) of soft food immediately after mixing (< 5 min) or after a resting time of 60 min + 35 ml fluid in infants or pre-school children with a resting gastric fluid pH of 1.8, mean of *n*=3 ± S.D.

**Fig. 7:**
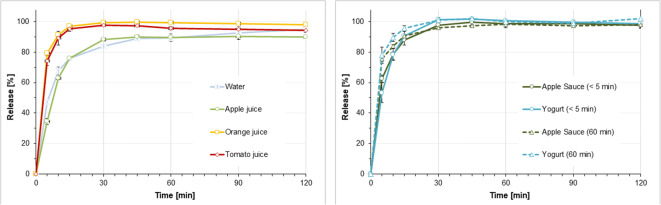
(**a**) Dissolution profiles obtained when simulating co-administration of granules containing 5 mg hydrocortisone with 1 teaspoon (5 ml) of fluid + 85 ml fluid in infants or pre-school children with a resting gastric fluid pH of 1.8, mean of *n*=3 ± S.D. (**b**) Dissolution profiles obtained when simulating co-administration granules containing 5 mg hydrocortisone with 1 teaspoon (5 ml) of soft food immediately after mixing (< 5 min) or after a resting time of 60 min + 85 ml fluid in infants or pre-school children with a resting gastric fluid pH of 1.8, mean of *n*=3 ± S.D.

**Fig. 8:**
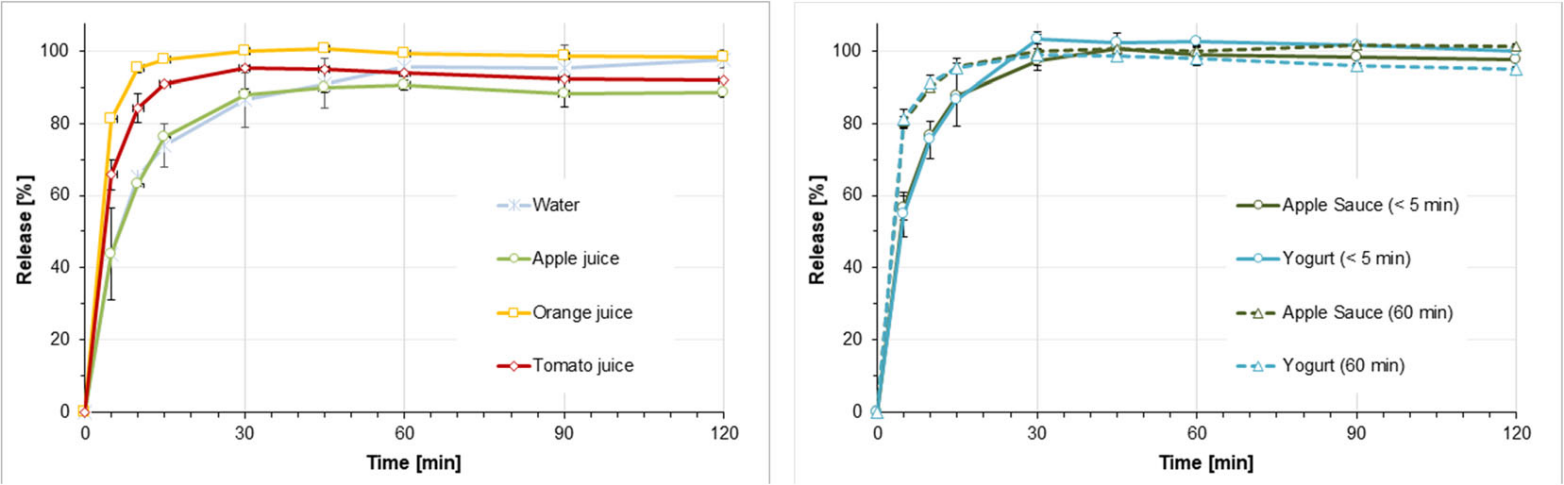
(**a**) Dissolution profiles obtained when simulating co-administration of granules containing 5 mg hydrocortisone with 1 teaspoon (5 ml) of fluid + 170 ml fluid school children with a resting gastric fluid pH of 1.8, mean of *n*=3 ± S.D. (**b**) Dissolution profiles obtained when simulating co-administration of granules containing 5 mg hydrocortisone with 1 teaspoon (5 ml) of soft food immediately after mixing (< 5 min) or after a resting time of 60 min + 170 ml fluid school children with a resting gastric fluid pH of 1.8, mean of *n*=3 ± S.D.

Figures 6 and 7 display the dissolution results obtained when simulating administration of hydrocortisone granules 2.5 mg with a teaspoon of fluid or soft food followed by ingestion of a small (35 ml) or a larger (85 ml) fluid volume to infants and pre-school children. Figure 8 displays the dissolution profiles observed when simulating administration of hydrocortisone granules 5 mg with a teaspoon of fluid or soft food followed by ingestion of a 170 ml fluid volume to school children.

When mimicking dose administration immediately after mixing with the dosing vehicle, dissolution of the 2.5 mg hydrocortisone dose in media and volumes intended to simulate initial gastric conditions after dose administration in infants and pre-school children was fast and complete, i.e. > 80% of the dose was released within 30 min (Figs. 6 and 7). This was independent of the dosing vehicle and the additional fluid volume applied. Similar observations were made in the release experiments mimicking administration of a 5 mg hydrocortisone dose in school children (Fig. 8). Moreover, in all media no drug precipitation or degradation could be observed over the entire test duration of 2 h. When comparing drug release profiles shown in Figs. 6a, 7a, 8a, when simulating co-administration with water and apple juice, the release rate was slightly slower than that in tomato juice and orange juice. Based on the visual observations made during the experiments, this is likely to be an artefact resulting from the high granule mass that had to be applied in the *in vitro* experiments to simulate administration of an individual dose. Due to the very low hydrocortisone load (0.66%) of the pellets, doses of 1.5 g, 0.75 g and 0.38 g granules per 200 ml release medium were required to address the three different dosing scenarios. Whereas due to the higher viscosity of tomato juice and orange juice, the granules were freely dispersed in the dissolution medium, coning was observed in the experiments with apple juice and water. The impact of coning was most pronounced when a granule dose of 1.5 g was used. Since *in vivo* hydrodynamics are different from those in a dissolution vessel, in this case, the paddle setup is unlikely to be an exact predictor of the *in vivo* release rate and the focus should be set on the complete dissolution within a short time range. Ultimately, we do not believe that this will make a significant difference in a real-world setting.

Simulation of dose administration 60 min after mixing with apple sauce or yogurt provided similar results, i.e. > 80% of the dose released within 30 min. However, a trend towards a slightly faster drug release compared to immediate administration after mixing with the dosing vehicle became visible. This might be a result of hydration of the hydrocortisone granules which can result in dissolution of part of the hydrocortisone dose during the 60 min resting time.

The dissolution results are in good agreement with the solubility data that indicated that there should be no solubility issues within the dose range administered to infants, pre-school children and school children. Overall, results from all dissolution experiments indicate that there should be no anticipated issues for the *in vivo* hydrocortisone release when the hydrocortisone granules are co-administered with common dosing vehicles such as water, apple-, orange- or tomato juice, apple sauce and yogurt. These observations are in good agreement with results from a recent clinical study where the hydrocortisone granules administered as sprinkles onto apple sauce or yogurt were bioequivalent to those administered directly to the back of the tongue (25).

### Chemical Compatibility/Stability of Hydrocortisone Granules and Dosing Vehicles

Since only those liquids and soft foods demonstrated to have no appreciable effect on drug product performance should be proposed as vehicles (14), a chemical compatibility/stability study was performed to support the observations made during the biorelevant dissolution experiments and to provide a complete *in vitro* risk assessment. Experiments were performed with both hydrocortisone standard and the hydrocortisone granules. The detailed results (Tables SII-SVII) are provided as supplementary material of this manuscript. Results of the studies demonstrated compatibility of drug and drug product with all dosing vehicles studied. After mixing the hydrocortisone granules with water or apple juice (Tables SII-SIII), very small concentrations of impurities B and G (all lower than the Ph.Eur. limit and also lower than the individual impurities limit of the USP could be observed after 60 min, but none of the other impurities were determined. In the mixtures of hydrocortisone reference with water or apple juice (Tables SII-SIII), no impurities could be detected. No impurities could be detected in samples from mixing granules or hydrocortisone reference with orange juice (Table SIV), tomato juice (Table SV), apple sauce (Table SVI) or yogurt (Table SVII) for 5 or 60 min. For some vehicles slight peak interferences (same retention times) of signals from the vehicle matrix (blank) and the peaks of some of the impurities in both the chromatograms for the hydrocortisone standard- and the hydrocortisone granule samples were observed. However, when comparing the peak areas obtained from the vehicle matrix alone with those obtained after mixing with granules or standards, it was obvious that there is no big difference, resulting in the conclusion that the peaks are caused by the vehicle matrix rather than by an impurity. This was supported by complete recovery of hydrocortisone (no change in potency) in the respective samples. Overall, results from the compatibility/stability study indicate compatibility of the proposed vehicles with the drug product.

## Conclusion

The results obtained in the present study confirm the compatibility and in-use chemical stability of hydrocortisone granules with/in commonly used dosing vehicles such as water, apple juice, orange juice, tomato juice, apple sauce and yogurt. In the simulated dosing scenarios applied in the dissolution experiments, *in vitro* dissolution was fast and complete, and no precipitation was observed over a test duration of 2 h, which would represent the maximum gastric residence time. Results of the chemical compatibility/stability study indicate that mixing with the set of dosing vehicles studied should not be an issue regarding degradation products. Overall, the present study results indicate that it is likely that *in vivo* dissolution of the hydrocortisone granules will not be affected by the composition of the co-administered fluids and soft foods studied. Since the studied dosing vehicles were shown to not alter performance of the drug product, they should be considered suitable for use as vehicles with the hydrocortisone granules.

## Electronic supplementary material

ESM 1 (DOCX 61 kb)

## ABBREVIATIONS

API: Active pharmaceutical ingredient
AI: Adrenal insufficiency
ACTH: Adrenocorticotropic hormone
EMA: European Medicines Agency
Ph.Eur.: European Pharmacopoeia
FDA: Food and Drug Administration
GI: Gastro-intestinal
IR: Immediate release
PIP: Paediatric Investigation Plan
PK: Pharmacokinetic
PTFE: Polytetrafluoroethylene
PVDF: Polyvinylidene fluoride
SGFsp: Simulated Gastric Fluid without pepsin
THF: Tetrahydrofuran
US: United States
USP: United States Pharmacopoeia
