# Supplementary material for: A Biopredictive In Vitro Approach for Assessing Compatibility of a Novel Pediatric Hydrocortisone Drug Product within Common Pediatric Dosing Vehicles
Source: Pharm Res. 2020 Sep 24;37(10):203. doi: 10.1007/s11095-020-02912-x (PMC7511475; doi:10.1007/s11095-020-02912-x)
Supplement: Supplementary file 1 — (DOCX 61 kb) [file 11095_2020_2912_MOESM1_ESM.docx]

**Supplementary material**

**Table SI: Energy and nutrient composition of the fluids and soft foods used in the *in vitro* study**

|  |  | Fluids | | | | | | | | | | Soft foods | | | | | |
| --- | --- | --- | --- | --- | --- | --- | --- | --- | --- | --- | --- | --- | --- | --- | --- | --- | --- |
|  |  | Water | Apple juice | | | Orange juice | | | Tomato juice | | | Apple sauce | | | Yoghurt | | |
|  |  | Humana | Tropicana | Alosa | Albi | Tropicana | Hohes C | Amecke | Rewe | Sonn-laender | Alnatura | MOTT´s | babylove | Lausitzer | Nestlé | Alpro | Weihen-stephan |
| Amount per | | - | 100 ml | 100 ml | 100 ml | 100 ml | 100 ml | 100 ml | 100 ml | 100 ml | 100 ml | 100 g | 100 g | 100 g | 100 g | 100 g | 100 g |
| Calories (kJ) | | - | 46 | 197 | 194 | 204 | 183 | 187 | 75 | 74 | 72 | 188 | 244 | 336 | 303 | 212 | 306 |
| Total fat (g) | | - | - | 0.1 | <0.5 | - | <0.5 | 0.1 | - | 0.1 | 0.10 | - | 0.6 | 0.5 | 3.5 | - | 3.5 |
| - saturated (g): | | - | - | <0.1 | <0.1 | - | <0.1 | 0.1 | - | 0.01 | 0.10 | - | 0.21 | - | 2.2 | 0.4 | - |
| Total carb. (g) | | - | 11.3 | 11.0 | 10.5 | 10.0 | 8.8 | 9.6 | 2.9 | 2.9 | 3.1 | 12 | 11.4 | 18 | 5.9 | 2.1 | 5.3 |
| - sugar (g): | | - | 10.4 | 10.5 | 10.2 | 10.0 | 8.8 | 9.6 | 2.9 | 2.9 | 2.8 | 10 | 10.3 | - | 5.6 | 2.1 | 5.3 |
| Dietary fibre (g) | | - | - | - | <0.5 | 0.7 | <1.0 | 0.4 | - | - | 0.2 | 1.8 | 2 | - | - | 1 | 0 |
| Protein (g) | | - | <0.4 | <0.1 | <0.1 | 0.8 | <1.0 | 0.5 | 0.8 | 0.7 | 1 | 0 | 0.3 | 0.3 | 4.5 | 4 | 4.4 |
| Salt (g) |  | - | - | <0.01 | <0.01 | 0 | 0.003 | 0.01 | 0.6 | 0.63 | 0.63 | - | - | - | 0.17 | 0.25 | 0.15 |
| Sodium (mg) | | - | 42 | - | - | - | - | - | - | - | - | - | - | - | - | - | - |
| Potassium (mg) | | - | 104 | - | - | - | - | - | - | - | - | 76.6 | - | - | - | - | - |
| Vitamin C (mg) | | - | - | - | - | 39.00 | 35.00 | - | - | - | - | 0.18 | - | - | - | - | - |

**Table SII: % Hydrocortisone and % impurities determined in water after mixing with hydrocortisone granules or hydrocortisone standard for 5 or 60 min (mean of n=3 ± S.D.)**

| **Pellets** | | | | | | | | | | |
| --- | --- | --- | --- | --- | --- | --- | --- | --- | --- | --- |
| **Time [min]** | **Hydrocortisone** | | **Impurity B (Cortisone)** | | **Impurity G** | | **pH** | | **Temperature [°C]** | |
|  | **%** | **S.D.** | **%** | **S.D.** | **%** | **S.D.** | **Start** | **End** | **Start** | **End** |
| 5 | 100.000 | 0.000 | 0.000 | 0.000 | 0.000 | 0.000 | 6.14 | 8.16 | 23.3 | 23.4 |
| 60 | 99.667 | 0.048 | 0.179 | 0.009 | 0.153 | 0.054 | 6.14 | 8.29 | 23.3 | 23.4 |
| **Hydrocortisone standard** | | | | | | | | | | |
| **Time [min]** | **Hydrocortisone** | | No impurities detectable | | | | **pH** | | **Temperature [°C]** | |
|  | **%** | **S.D.** |  |  |  |  | **%** | **S.D.** | **%** | **S.D.** |
| 5 | 100.000 | 0.000 |  |  |  |  | 6.14 | 5.90 | 23.3 | 23.4 |
| 60 | 100.000 | 0.000 |  |  |  |  | 6.14 | 5.88 | 23.3 | 23.4 |

**Table SIII: % Hydrocortisone and % impurities determined in apple juice after mixing with hydrocortisone granules or hydrocortisone standard for 5 or 60 min (mean of n=3 ± S.D.)**

| **Pellets** | | | | | | | | | | |
| --- | --- | --- | --- | --- | --- | --- | --- | --- | --- | --- |
| **Time [min]** | **Hydrocortisone** | | **Impurity B (Cortisone)** | | **Impurity G** | | **pH** | | **Temperature [°C]** | |
|  | **%** | **S.D.** | **%** | **S.D.** | **%** | **S.D.** | **Start** | **End** | **Start** | **End** |
| 5 | 99.804 | 0.170 | 0.196 | 0.170 | 0.000 | 0.000 | 3.75 | 3.82 | 24.7 | 24.4 |
| 60 | 99.618 | 0.063 | 0.337 | 0.032 | 0.045 | 0.039 | 3.75 | 3.77 | 24.7 | 24.4 |
| **Hydrocortisone standard** | | | | | | | | | | |
| **Time [min]** | **Hydrocortisone** | | No impurities detectable | | | | **pH** | | **Temperature [°C]** | |
|  | **%** | **S.D.** |  |  |  |  | **%** | **S.D.** | **%** | **S.D.** |
| 5 | 100.000 | 0.000 |  |  |  |  | 3.75 | 3.64 | 24.7 | 24.4 |
| 60 | 100.000 | 0.000 |  |  |  |  | 3.75 | 3.65 | 24.7 | 24.4 |

**Table SIV: % Hydrocortisone and % impurities determined in orange juice after mixing with hydrocortisone granules or hydrocortisone standard for 5 or 60 min (mean of n=3 ± S.D.)**

| **Pellets** | | | | | | | |
| --- | --- | --- | --- | --- | --- | --- | --- |
| **Time [min]** | **Hydrocortisone** | | No impurities detectable* | **pH** | | **Temperature [°C]** | |
|  | **%** | **S.D.** |  | **Start** | **End** | **Start** | **End** |
| 5 | 100.000 | 0.000 |  | 3.87 | 3.90 | 22.4 | 22.9 |
| 60 | 100.000 | 0.000 |  | 3.87 | 4.01 | 22.4 | 22.4 |
| **Hydrocortisone standard** | | | | | | | |
| **Time [min]** | **Hydrocortisone** | | No impurities detectable* | **pH** | | **Temperature [°C]** | |
|  | **%** | **S.D.** |  | **%** | **S.D.** | **%** | **S.D.** |
| 5 | 100.000 | 0.000 |  | 3.87 | 3.87 | 22.4 | 22.9 |
| 60 | 100.000 | 0.000 |  | 3.87 | 3.93 | 22.4 | 22.4 |

**Table SV: % Hydrocortisone and % impurities determined in tomato juice after mixing with hydrocortisone granules or hydrocortisone standard for 5 or 60 min (mean of n=3 ± S.D.)**

| **Pellets** | | | | | | | |
| --- | --- | --- | --- | --- | --- | --- | --- |
| **Time [min]** | **Hydrocortisone** | | No impurities detectable* | **pH** | | **Temperature [°C]** | |
|  | **%** | **S.D.** |  | **Start** | **End** | **Start** | **End** |
| 5 | 100.000 | 0.000 |  | 4.05 | 4.10 | 22.4 | 22.9 |
| 60 | 100.000 | 0.000 |  | 4.05 | 4.05 | 22.4 | 23.7 |
| **Hydrocortisone standard** | | | | | | | |
| **Time [min]** | **Hydrocortisone** | | No impurities detectable* | **pH** | | **Temperature [°C]** | |
|  | **%** | **S.D.** |  | **%** | **S.D.** | **%** | **S.D.** |
| 5 | 100.000 | 0.000 |  | 4.05 | 4.05 | 22.4 | 22.9 |
| 60 | 100.000 | 0.000 |  | 4.05 | 4.07 | 22.4 | 24.1 |

**Table SVI: % Hydrocortisone and % impurities determined in apple sauce after mixing with hydrocortisone granules or hydrocortisone standard for 5 or 60 min (mean of n=3 ± S.D.)**

| **Pellets** | | | | | | | |
| --- | --- | --- | --- | --- | --- | --- | --- |
| **Time [min]** | **Hydrocortisone** | | No impurities detectable* | **pH** | | **Temperature [°C]** | |
|  | **%** | **S.D.** |  | **Start** | **End** | **Start** | **End** |
| 5 | 100.000 | 0.000 |  | 3.28 | 3.20 | 23.0 | 22.5 |
| 60 | 100.000 | 0.000 |  | 3.28 | 3.15 | 23.0 | 22.3 |
| **Hydrocortisone standard** | | | | | | | |
| **Time [min]** | **Hydrocortisone** | | No impurities detectable* | **pH** | | **Temperature [°C]** | |
|  | **%** | **S.D.** |  | **%** | **S.D.** | **%** | **S.D.** |
| 5 | 100.000 | 0.000 |  | 3.28 | 3.87 | 23.0 | 23.0 |
| 60 | 100.000 | 0.000 |  | 3.28 | 3.93 | 23.0 | 23.8 |

**Table SVII: % Hydrocortisone and % impurities determined in yoghurt after mixing with hydrocortisone granules or hydrocortisone standard for 5 or 60 min (mean of n=3 ± S.D.)**

| **Pellets** | | | | | | | |
| --- | --- | --- | --- | --- | --- | --- | --- |
| **Time [min]** | **Hydrocortisone** | | No impurities detectable* | **pH** | | **Temperature [°C]** | |
|  | **%** | **S.D.** |  | **Start** | **End** | **Start** | **End** |
| 5 | 100.000 | 0.000 |  | 4.17 | 4.15 | 23.0 | 22.5 |
| 60 | 100.000 | 0.000 |  | 4.17 | 4.13 | 23.0 | 22.4 |
| **Hydrocortisone standard** | | | | | | | |
| **Time [min]** | **Hydrocortisone** | | No impurities detectable* | **pH** | | **Temperature [°C]** | |
|  | **%** | **S.D.** |  | **%** | **S.D.** | **%** | **S.D.** |
| 5 | 100.000 | 0.000 |  | 4.17 | 4.13 | 23.0 | 23.0 |
| 60 | 100.000 | 0.000 |  | 4.17 | 4.10 | 23.0 | 22.6 |
